# Stanniocalcin-1 Overexpression Protects Porcine Intestinal Epithelial Cells Against TBHP-Induced Oxidative Stress by Preserving Mitochondrial Homeostasis

**DOI:** 10.3390/ani16172668

**Published:** 2026-08-25

**Authors:** Liming Wu, Yan Bin, Yubei Wei, Zihan Cui, Jiayi Du, Jinluo Lv, Xiaoliang Xiang

**Affiliations:** 1 Hunan Provincial Higher Education Key Laboratory of Intensive Processing Research on Mountain Ecological Food, College of Biological and Food Engineering, Huaihua University, Huaihua 418000, China; wlm@hhtc.edu.cn (L.W.); bf_bbbyy@163.com (Y.B.); ooorange_leo@163.com (Y.W.); 15122454286@163.com (Z.C.); 17708458520@163.com (J.D.); m13147780105@163.com (J.L.); 2 Key Laboratory of Research and Utilization of Ethnomedicinal Plant Resources of Hunan Province, Huaihua University, Huaihua 418000, China

**Keywords:** stanniocalcin-1, IPEC-J2 cells, oxidative stress, mitophagy, AMPK/Nrf2/Sirt1 pathway

## Abstract

Stanniocalcin-1 (STC-1) is a multifunctional glycoprotein; however, its role in protecting intestinal epithelial cells against oxidative injury has not been completely elucidated. This study investigated the cytoprotective effects and underlying molecular mechanisms of STC-1 overexpression in porcine intestinal epithelial (IPEC-J2) cells subjected to tert-butyl hydroperoxide (TBHP)-induced oxidative stress. IPEC-J2 cells were transfected with pcDNA3.1/STC-1 prior to TBHP challenge. STC-1 overexpression markedly rescued cells from TBHP-induced cytotoxicity and cell death, and was associated with a reduced Bax/Bcl-2 ratio. Concurrently, elevated STC-1 expression dramatically suppressed intracellular reactive oxygen species and mitochondrial superoxide accumulation while preserving the mitochondrial membrane potential. These physiological improvements were accompanied by enhanced total antioxidant capacity and activities of key antioxidant enzymes. Mechanistically, STC-1 overexpression enhanced autophagic flux and promoted Pink1/Parkin-mediated mitophagy to eliminate dysfunctional mitochondria. Furthermore, STC-1 upregulation potentiated the AMPK–Nrf2/Sirt1 signaling axis and the subsequent transcriptional upregulation of mitochondrial quality control markers, including FoxO1, PGC-1α, and TFAM, under stress conditions. Collectively, these findings demonstrate that STC-1 safeguards porcine intestinal cells against oxidative injury by orchestrating a cooperative defense network encompassing ROS scavenging, mitochondrial homeostasis, and antioxidant defense amplification via the AMPK–Nrf2/Sirt1 pathway, highlighting a potential therapeutic target for preventing stress-associated intestinal disorders in piglets.

## Simple Summary

Oxidative stress is an important factor that affects intestinal health and growth performance in young animals. This study investigated whether stanniocalcin-1, an endogenous cytoprotective protein, could alleviate oxidative injury in porcine intestinal cells. Our results demonstrated that increasing stanniocalcin-1 production improved the ability of cells to resist stress, reduced cellular damage, preserved the function of energy-producing structures, and enhanced the removal of damaged cellular components. Collectively, these findings provide novel insights into how intestinal cells protect themselves from injury and suggest that stanniocalcin-1 may be useful for improving animal health and stress resistance in livestock production.

## 1. Introduction

In pig production, the weaning period is a critical stage during which piglets are highly susceptible to various stressors due to abrupt separation from the sow, dietary transitions, and environmental changes [1]. These alterations can induce the body to generate excessive reactive oxygen species (ROS), which can disrupt redox homeostasis and induce oxidative damage and mitochondrial dysfunction [2]. Given the immature immune and antioxidant systems of weaned piglets, oxidative stress contributes to poor growth performance and increased disease susceptibility [3]. Therefore, maintaining redox balance is essential for intestinal health.

Mitochondria are both a major source and target of ROS, playing a pivotal role in oxidative stress-induced intestinal injury [4]; excessive ROS levels disrupt mitochondrial function, induce lipid peroxidation [5], and trigger mitochondria-mediated cell death. To protect cells from such oxidative damage, cells activate mitochondrial quality control mechanisms, comprising mitophagy [6] and mitochondrial biogenesis, alongside enzymatic antioxidant defense systems, such as catalase (CAT), glutathione peroxidase (GSH-Px), superoxide dismutase (SOD), and total antioxidant capacity (T-AOC) [7]. Specifically, damaged mitochondria are selectively eliminated via Pink1/Parkin-mediated mitophagy to limit ROS accumulation [8,9]. Concurrently, mitochondrial biogenesis is stimulated via peroxisome proliferator-activated receptor-γ coactivator-1α (PGC-1α) and its downstream target, mitochondrial transcription factor A (TFAM) [10], to replenish healthy organelles. Central to coordinating this entire network is AMP-activated protein kinase (AMPK), which is an energy sensor that promotes nuclear respiratory factor-1/2 (Nrf1/2)-mediated antioxidant transcription [11]. Upon activation, AMPK cooperates with Sirtuin 1 (Sirt1) to regulate PGC-1α- and Forkhead Box O1 (FoxO1)-dependent mitochondrial biogenesis and autophagy [4], thereby restoring mitochondrial homeostasis and improving cellular resistance to oxidative stress.

Accordingly, the identification of novel endogenous regulators of antioxidant defenses may offer new strategies for improving intestinal health in piglets, alongside the well-documented antioxidant benefits of various conventional substances [12,13] in pig production. STC-1 is the mammalian homolog of fish stanniocalcin and exerts multiple functions in mammals, including mineral metabolism [14]. STC-1 has been reported to exhibit cytoprotective effects in multiple tissues by suppressing ROS generation [15], preserving mitochondrial function [16], maintaining cellular bioenergetics [17], and inhibiting apoptosis [18] in various animal [15,19,20] and cell models [21,22,23]. However, its function in porcine intestinal epithelial cells under oxidative stress is largely unknown. In particular, the potential involvement of STC-1 in maintaining mitochondrial homeostasis via coordinated regulation of mitochondrial quality control, including mitophagy and mitochondrial biogenesis, has not been systematically investigated.

Unlike previous studies that primarily focused on the antioxidant or cytoprotective properties of STC-1, the present study aimed to investigate whether STC-1 coordinates multiple aspects of mitochondrial quality control in porcine IPEC cells during oxidative stress. Therefore, this study assessed the effects of STC-1 overexpression on antioxidant capacity, mitochondrial function, mitophagy, mitochondrial biogenesis, and the AMPK–Nrf2/Sirt1 signaling pathway in TBHP-challenged IPEC-J2 cells. By integrating these responses, this study provides new insights into the potential protective role of STC-1 in maintaining mitochondrial homeostasis during oxidative stress.

## 2. Materials and Methods

### 2.1. Cell Culture

Porcine intestinal epithelial cells (IPEC-J2 cells), derived from DSMZ (ACC-701), were purchased from Procell System (Wuhan, China). The cells were cultured in DMEM/F12 medium (Hyclone, Logan, UT, USA) supplemented with 10% fetal bovine serum (Gibco, Carlsbad, CA, USA), 100 U/mL penicillin, and 100 µg/mL streptomycin (Beyotime, Shanghai, China) at 37 °C in a humidified atmosphere containing 5% CO_2_.

### 2.2. Construction of STC1 Expression Vector

Total RNA was extracted from IPEC-J2 cells using Trizol reagent (Invitrogen, Carlsbad, CA, USA) and converted to cDNA using a Synthesis Kit (TaKaRa, Dalian, China). The full CDS of porcine *STC-1* was amplified using the following primers: 5′-GAGCTCGGATCCATGCTCCAAAACTCAGCAG-3′ (sense) and 5′-TCTAGACTCGAGTTACGCACTCTCGTGGGAG-3′ (antisense). Purified amplicons were cloned into pcDNA3.1(+) (Invitrogen) between the *BamHI* and *XhoI* restriction sites using T4 DNA ligase (TaKaRa). The resulting recombinant plasmid was designated as pcDNA3.1/STC-1. Endotoxin-free plasmid DNA was extracted from an overnight culture of competent cells harboring pcDNA3.1/STC-1 and pcDNA3.1(+) using a commercial kit (D6926-03, Omega, Doraville, GA, USA).

### 2.3. Transfection and Treatment of IPEC-J2 Cells

The cells were seeded in 6-well plates (2 × 10^5^ cells/well) and cultured to 60–70% confluence. The cells were transfected with 2.5 µg/well of pcDNA3.1/STC-1 or empty pcDNA3.1(+) vector using Lipofectamine 3000 (Invitrogen). After 48 h of incubation with either the plasmids, the positive antioxidant control N-acetylcysteine (NAC, Omega), or a vehicle (DMSO, Beyotime), the cells were thoroughly washed with PBS followed by exposure to 400 μM TBHP (75-91-2, Sigma-Aldrich, St. Louis, MO, USA) or an equal volume of PBS for 4 h. Finally, cell morphology and viability were assessed via phase-contrast microscopy (IX51, Olympus, Tokyo, Japan) and the MTT assay, respectively.

For subsequent experiments, the cells were randomly assigned to four groups: pcDNA3.1(+) + PBS, pcDNA3.1/STC-1 + PBS, pcDNA3.1(+) + TBHP, and pcDNA3.1/STC-1 + TBHP. Following 48 h of incubation after plasmid transfection, the cells were exposed to TBHP for 4 h. Specifically, 400 μM TBHP was used for morphological observation and cell viability/death assessment, and 200 μM TBHP was used for other measurements. For Nrf2 detection, 10 μM MG-132 (HY-13259, MCE, Monmouth Junction, NJ, USA) was also added to the culture medium with TBHP or PBS, and then incubated for 4 h to prevent proteasomal degradation. Then, the cells, cell lysates, and culture supernatants were collected for subsequent analyses.

### 2.4. Measurement of Cell Viability

The cells were seeded in 96-well plates at a density of 1 × 10^4^ cells/well and treated as detailed above. Following the treatments, the cells were incubated with 10 μM MTT (Beyotime) for an additional 4 h. The medium was then replaced with 100 µL of DMSO, and the plates were shaken for 10 min. The absorbance at 490 nm was measured using a microplate reader (Epoch 2, BioTek, Winooski, VT, USA). Cell viability was calculated and normalized against the control group.

The cell viability status was visualized using an Acridine Orange and Propidium Iodide (AO/PI) dual-fluorescence staining kit (ST1569, Beyotime) according to the manufacturer’s instructions. Briefly, after the removal of the culture medium, the cells were washed with PBS and incubated with the AO/PI working solution in the dark for 10 min. Then, fluorescence images were then captured using a fluorescence microscope (IX73, Olympus) under 488 nm excitation. Five random fields of view per sample were photographed for cell counting. The percentage of PI-positive cells was calculated relative to the total cell count.

### 2.5. Assessment of Intracellular ROS and Mitochondrial Superoxide Levels

Following the indicated treatments, the intracellular ROS levels were assessed using a 2′,7′-dichlorofluorescein diacetate probe (DCFH-DA probe; S1105S, Beyotime), followed by incubation in the dark for 30 min. After washing with PBS, green fluorescence was recorded using a fluorescence microscope (IX71, Olympus) at an excitation wavelength of 488 nm. To detect mitochondrial superoxide levels, the cells were incubated with 5 μM MitoSOX Red (HY-D1055, MCE) at 37 °C for 20 min. The nuclei were counterstained with Hoechst 33342 (C1029, Beyotime), and fluorescence images were captured using excitation wavelengths of 350 nm (Hoechst) and 510 nm (MitoSOX), respectively. Fluorescence levels were quantified via ImageJ software (version 1.54g) and expressed as percentages relative to the control group.

### 2.6. Biochemical Assays

Culture supernatants of the treated cells were collected after centrifugation for the measurement of MDA, NO, and LDH contents, while cell lysates were harvested via ultrasonication [14] for T-AOC and antioxidant enzyme activity assays. The protein concentrations were measured using a BCA kit (P0009, Beyotime) for normalization. The MDA content was determined by the thiobarbituric acid colorimetric method based on OD values at 450, 532, and 600 nm, and then calculated as μmol/mg of protein. Nitric oxide (NO) levels were determined using the nitrate reductase method (A012-1, Jiancheng, Nanjing, China) and expressed as μmol/mg of protein. Lactate dehydrogenase (LDH) release was assayed using a commercial kit (C0016, Beyotime) according to the manufacturer’s instructions, with all values normalized to the maximum LDH release group.

The T-AOC in cells was measured using the ABTS method with a commercial kit (A015-2-1, Jiancheng) following the manufacturer’s instructions. The results were expressed as mM Trolox equiv/L. The total SOD (A001-3, Jiancheng), GSH-Px (A005-1, Jiancheng), and CAT (A007-1, Jiancheng) activities were measured following the manufacturer’s protocols. The unit for enzyme activity is U/mg of protein.

### 2.7. Measurement of Mitochondrial Membrane Potential (MMP)

MMP was evaluated using a JC-1 fluorescent probe (C2005, Beyotime) according to the manufacturer’s instructions. Briefly, the cells were incubated with the JC-1 working solution in the dark. Fluorescence was captured at excitation wavelengths of 488 nm (green fluorescence, representing JC-1 monomers/decreased MMP) and 525 nm (red fluorescence, representing JC-1 aggregates/normal MMP). The relative MMP was quantified using ImageJ software and expressed as the red-to-green fluorescence intensity ratio.

### 2.8. Autophagy Flux Detection

Cellular autophagic flux was monitored using the mRFP-GFP-LC3 dual-fluorescence labeling method. Briefly, following the indicated treatments, IPEC-J2 cells were detached and collected via centrifugation at 1000 rpm for 10 min. Cell pellets were resuspended in an Ad-mRFP-GFP-LC3 adenovirus solution (AP22102709, HanBio, Shanghai, China) containing 2 μg/mL polybrene. After a 15 min incubation at room temperature, the cells were transferred onto coverslips in 12-well plates and infected for an additional 2 h at 37 °C, followed by overnight incubation in fresh growth medium. Subsequently, the cells were fixed with 4% paraformaldehyde and rinsed with PBST. The nuclei were counterstained with Hoechst 33342 for 5 min, followed by a thorough wash with PBST. Autophagy was visualized using a confocal laser scanning microscope (FV3000, Olympus). Autophagic flux was quantified by evaluating the GFP and RFP puncta from five randomly selected fields of view per sample. Yellow puncta (RFP^+^ + GFP^+^) represented autophagosomes, whereas red-only puncta (RFP^+^ + GFP^−^) indicated autolysosomes.

### 2.9. Western Blot

Cell lysates were prepared using ice-cold RIPA lysis buffer (P0013B, Beyotime) supplemented with 1 mM PMSF (G2008, Servicebio, Wuhan, China), and a protease and phosphatase inhibitor cocktail (G2006 and G2007, Servicebio). For Nrf2 expression analysis, 10 μM MG-132 (HY-13259, MCE) was also included in the lysis buffer to preserve Nrf2 protein levels. After centrifugation at 14,000× *g*, the supernatants were collected, and protein concentrations were determined. A 20 μg amount of protein from each sample was separated via SDS-PAGE on 12% or 15% gels and transferred to a PVDF membrane (Millipore, Bedford, MA, USA). Following blocking with 5% (*w*/*v*) non-fat milk, the membranes were incubated overnight with diluted rabbit antibodies (listed in Table 1). Immunoreactive bands were visualized using an enhanced chemiluminescence substrate and the band intensities were quantified using densitometry and an automatic analyzer (Tanon-5200, Shanghai, China). All target protein signals were normalized to the corresponding loading controls as indicated in figures.

### 2.10. Statistical Analysis

All experiments were performed with at least three independent biological replicates, and the data are presented as the mean ± SD in all figures. Statistical analysis was performed using one-way ANOVA followed by Tukey’s multiple comparisons test. A *p*-value < 0.05 was considered statistically significant. Statistical analyses were performed and figures were constructed using Graph Pad Prism 10.0 (San Diego, CA, USA).

## 3. Results

### 3.1. STC-1 Overexpression Attenuates TBHP-Induced Cell Death in IPEC-J2 Cells

**Figure 1:**
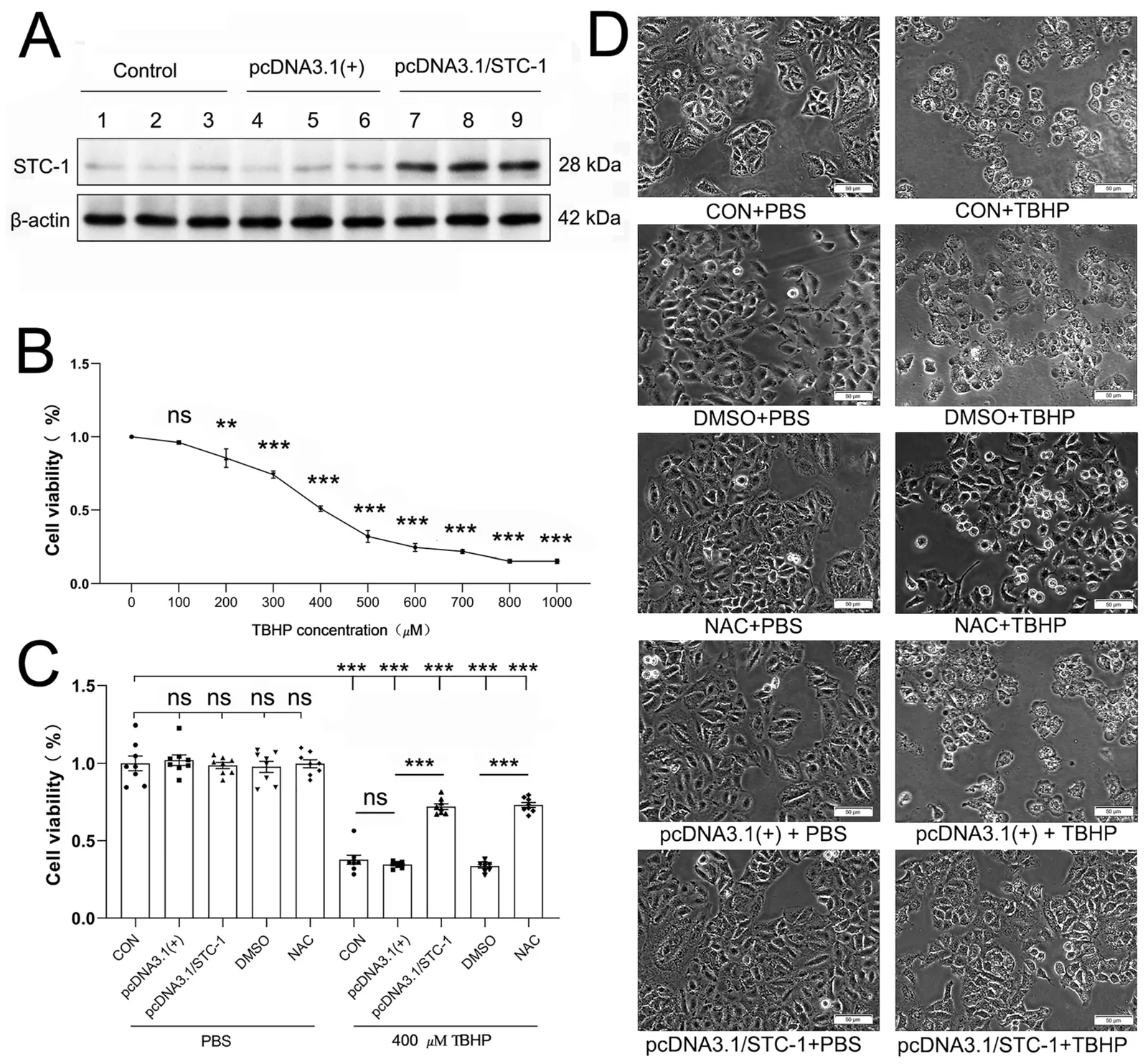
STC-1 overexpression protects IPEC-J2 cells against TBHP-induced oxidative injury. (**A**) Western blot analysis of STC-1 protein expression in cells 48 h post-transfection. (**B**) Viability of IPEC-J2 cells exposed to gradient concentrations of TBHP for 4 h, as determined by the MTT assay. (**C**) Assessment of the effects of STC-1 overexpression on cell viability under oxidative stress. Cells were transfected with pcDNA3.1/STC-1 or the empty vector for 48 h prior to a 4 h exposure to 400 μM TBHP. NAC and DMSO served as the positive antioxidant and vehicle controls, respectively. (**D**) Representative phase-contrast images showing morphological changes in cells exposed to the indicated treatments (scale bar = 50 μm). Data are presented as the mean ± SD (*n* = 6) of six independent experiments. Different numbers of asterisks indicate significant differences among groups (** *p* < 0.01, *** *p* < 0.001; ns, *p* > 0.05 vs. the indicated group).

After 48 h of transfection, STC-1 protein expression was markedly increased in IPEC-J2 cells transfected with pcDNA3.1/STC-1 (Figure 1A). Exposure to 200 and 400 μM TBHP for 4 h reduced cell viability to approximately 80% and 51% of the control level, respectively (Figure 1B). Therefore, 400 μM was used for cell death-related assessments as it induced clear oxidative injury but the cells retained sufficient cell integrity for quantitative analysis; a non-lethal concentration of 200 μM was used for biochemistry and other assays to avoid excessive cell detachment.

The MTT assay results showed that STC-1 overexpression significantly attenuated TBHP-induced cell death, maintaining cell viability at approximately 72% of the control level, whereas viability in the empty vector group was only about 35% (*p* < 0.001). Similarly, NAC significantly alleviated TBHP-induced cytotoxicity compared with the DMSO-treated group (Figure 1C). Consistent with the cell viability results, phase-contrast microscopy showed that STC-1-overexpressing and NAC-treated cells largely retained normal morphology and remained attached after 400 μM TBHP exposure, whereas cells transfected with the empty vector or treated with the vehicle exhibited marked cell shrinkage, rounding, and detachment (Figure 1D).

**Figure 2:**
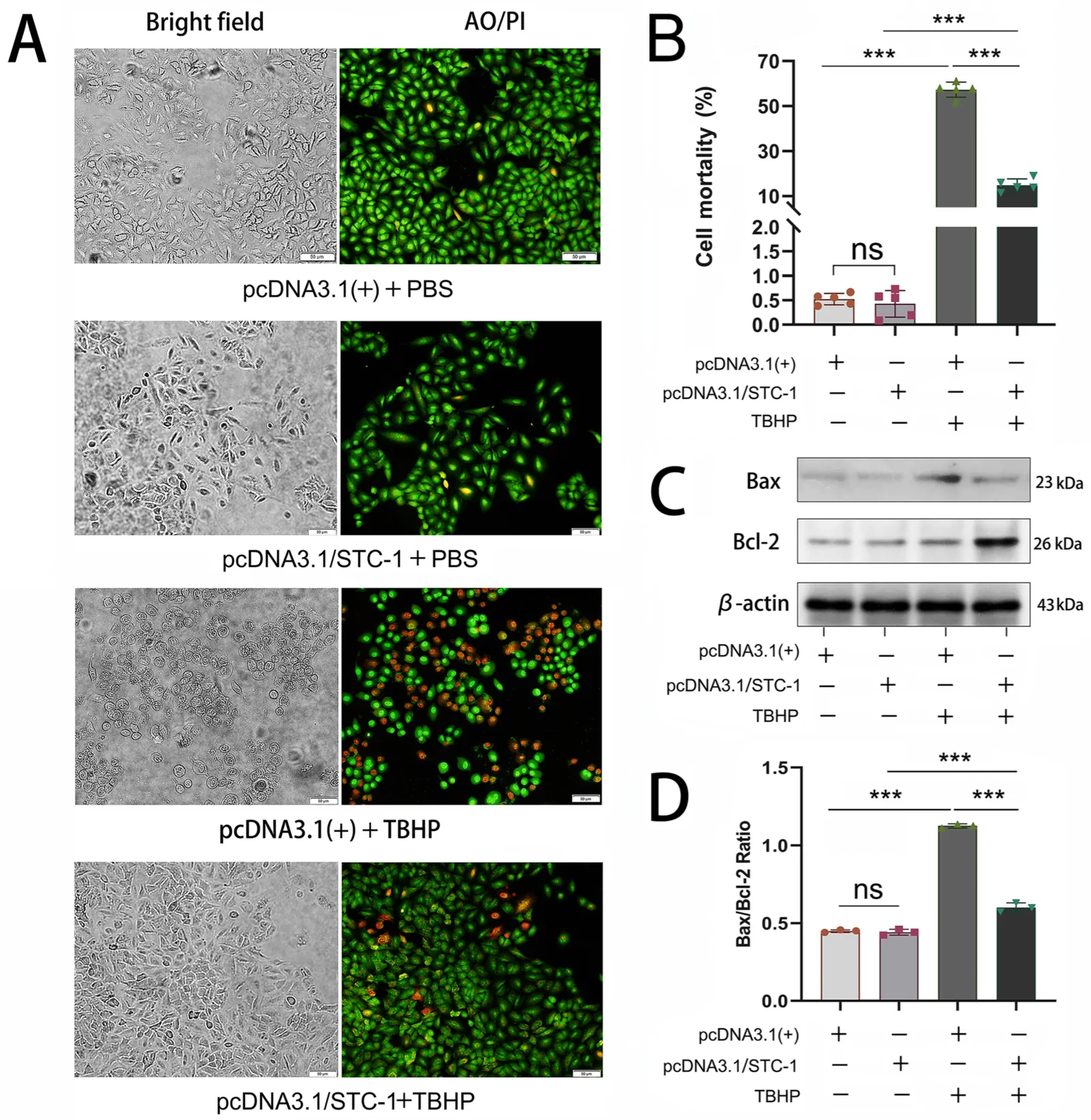
STC-1 overexpression attenuates TBHP-induced viability declines and modulates apoptosis-related signaling in IPEC-J2 cells. Cells transfected with pcDNA3.1(+) or pcDNA3.1/STC-1 were challenged with 400 μM TBHP for 4 h. (**A**) Representative bright-field and AO/PI dual-fluorescence micrographs illustrating cell morphology and viability status (scale bar = 50 μm). Live cells exhibit green fluorescence (AO-positive), whereas cells with compromised membrane integrity show orange/red fluorescence (PI-positive). (**B**) Quantitative analysis of the percentage of PI-positive cells. (**C**,**D**) Western blot analysis and densitometric quantification of Bax and Bcl-2 expression levels. Data are presented as the mean ± SD (*n* = 3 or 5) of three or six independent experiments. *** *p* < 0.001; ns, *p* > 0.05 vs. the indicated group.

Moreover, AO/PI staining further demonstrated that TBHP treatment significantly increased the proportion of PI-positive cells in the pcDNA3.1(+) group, whereas STC-1 overexpression significantly attenuated this increase (all *p* < 0.001, Figure 2A,B). To further examine apoptosis-related proteins, Western blotting was performed, which revealed that TBHP treatment markedly increased the Bax/Bcl-2 ratio in pcDNA3.1(+)-transfected cells (Figure 2C), whereas STC-1 overexpression restored the balance by decreasing Bax and increasing Bcl-2 expression (*p* < 0.001, Figure 2D).

### 3.2. STC-1 Overexpression Confers Resistance to Oxidative Stress and Enhances the Activity of Antioxidant Enzymes

**Figure 3:**
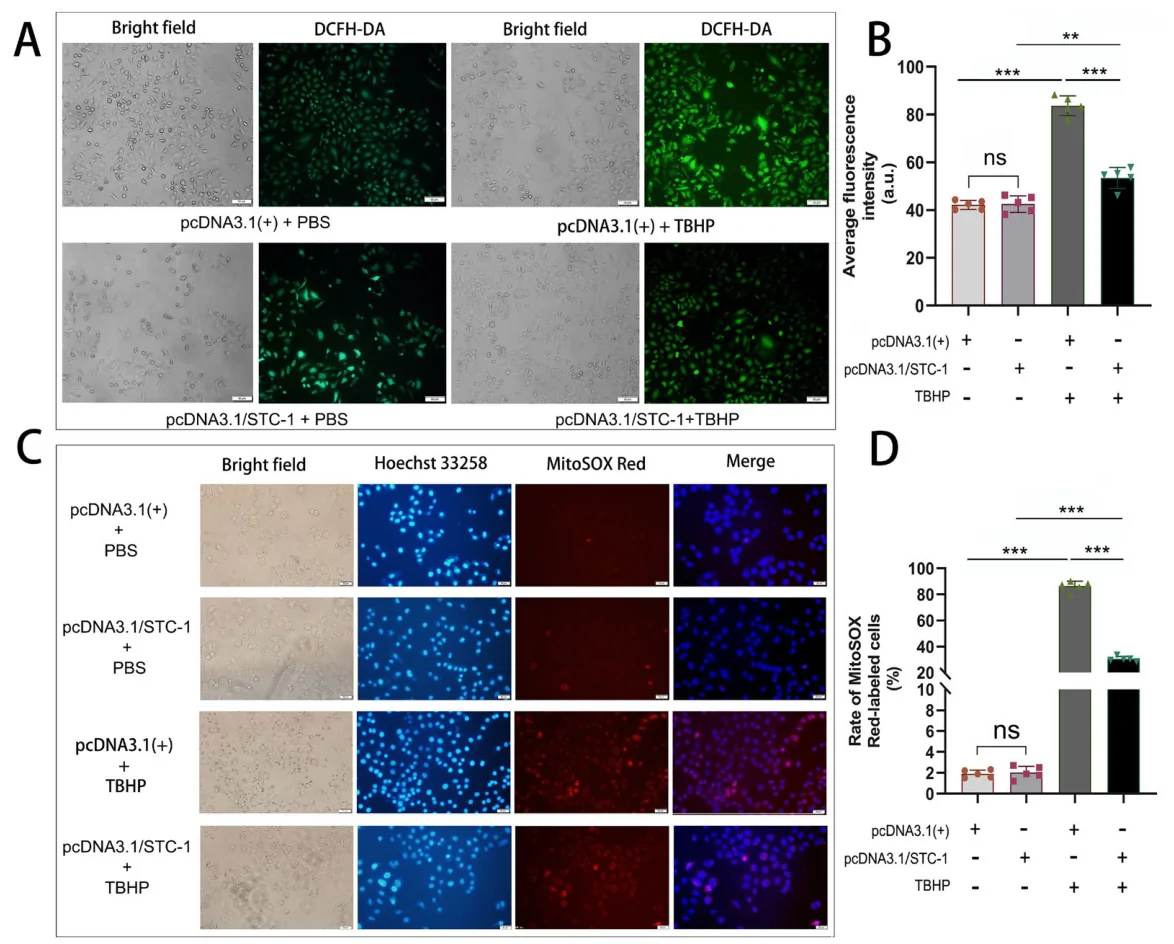
STC-1 overexpression suppresses intracellular ROS and mitochondrial superoxide production in TBHP-treated IPEC-J2 cells. (**A**) Representative bright-field and DCFH-DA fluorescence micrographs illustrating intracellular ROS levels in cells transfected with pcDNA3.1/STC-1 or the empty vector for 48 h, followed by a 4 h challenge with 200 μM TBHP or PBS. (**B**) Quantitative analysis of DCF fluorescence intensity. (**C**) Representative images of mitochondrial superoxide accumulation detected via MitoSOX Red staining, with nuclei counterstained using Hoechst 33342. (**D**) Quantification of the percentage of MitoSOX Red-positive cells. Data are presented as the mean ± SD (*n* = 5). Statistical significance is indicated in the figure. ** *p* < 0.001, *** *p* < 0.001; ns, *p* > 0.05 vs. the indicated group. Scale bar = 50 μm.

Intracellular ROS and mitochondrial superoxide levels were detected using DCFH-DA and MitoSOX Red staining, respectively. As shown in Figure 3A,B, STC-1 overexpression did not significantly affect basal ROS levels. However, 200 μM TBHP markedly increased intracellular ROS in pcDNA3.1(+)-transfected cells (*p* < 0.001). In contrast, STC-1 overexpression significantly suppressed TBHP-induced ROS accumulation, as demonstrated by the pronounced reduction in green DCF fluorescence intensity (*p* < 0.001 vs. pcDNA3.1(+) + TBHP). Similarly, MitoSOX Red staining showed minimal mitochondrial superoxide production in both transfection groups without TBHP treatment (Figure 3C,D). TBHP exposure dramatically increased mitochondrial superoxide levels, with more than 85% of pcDNA3.1(+)-transfected cells being MitoSOX-positive (*p* < 0.001 vs. PBS). In contrast, STC-1 overexpression significantly attenuated mitochondrial superoxide accumulation (*p* < 0.001 vs. pcDNA3.1(+) + TBHP), reducing the percentage of MitoSOX-positive cells to approximately 30%.

**Figure 4:**
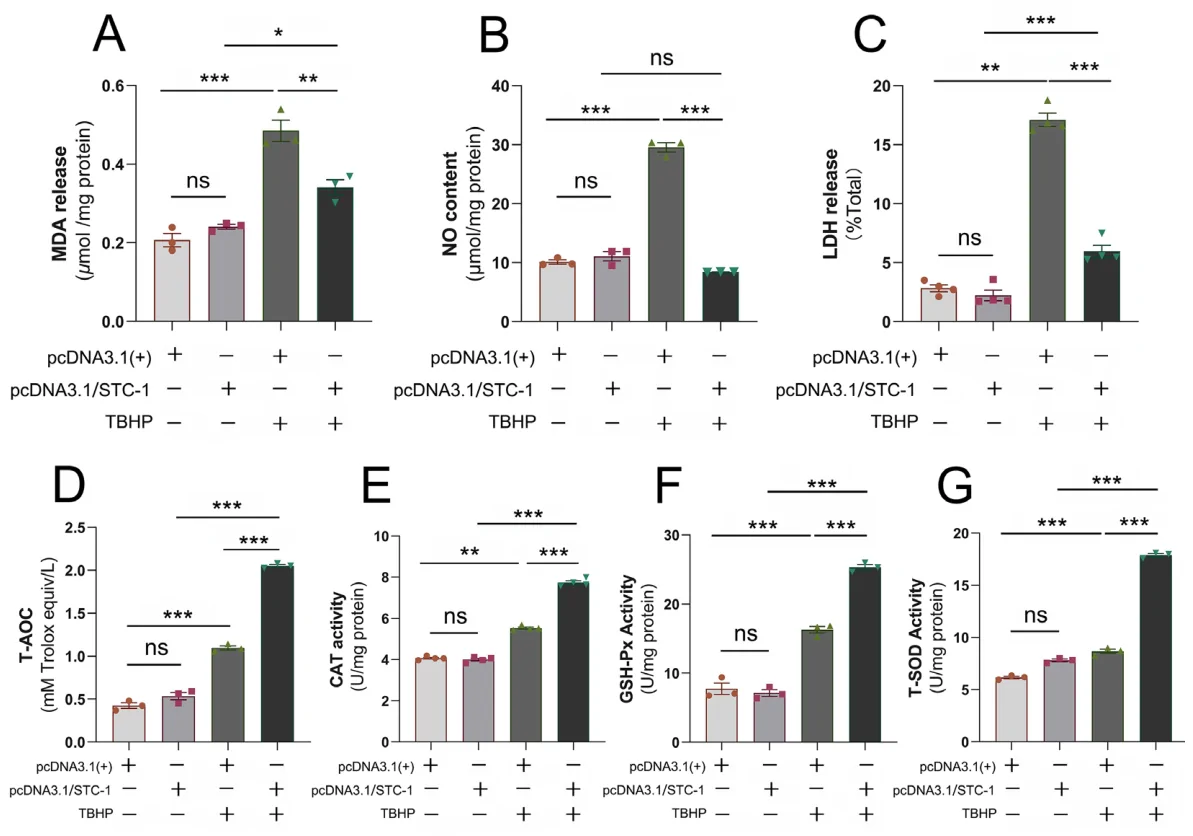
STC-1 overexpression alleviates TBHP-induced oxidative injury and enhances antioxidant defense in IPEC-J2 cells. Transfected cells were cultured in the presence or absence of 200 μM TBHP for 4 h. Subsequently, biochemical assays were performed to evaluate the following: biomarkers of oxidative damage: (**A**) MDA content, (**B**) NO content, and (**C**) LDH release; antioxidant parameters: (**D**) T-AOC, (**E**) CAT, (**F**) GSH-Px, and (**G**) T-SOD activities. Data are presented as the mean ± SD (*n* = 3). Each column represents a triplicate experiment. Statistical significance is indicated within the panels (* *p* < 0.05, ** *p* < 0.01, and *** *p* < 0.001; ns, *p* > 0.05).

The assessment of oxidative stress-associated biochemical parameters showed that PBS treatment did not significantly affect the parameters in the transfection groups. In contrast, TBHP exposure elicited pronounced increases in MDA accumulation, NO content, and LDH release (all *p* < 0.01) in the pcDNA3.1(+) group. However, STC-1 overexpression significantly suppressed MDA accumulation, NO production, and LDH release (Figure 4A–C, all *p* < 0.01 vs. vector + TBHP group).

The cellular antioxidant capacity evaluations are presented in Figure 4D–G. No significant differences were observed between the two transfection groups without TBHP treatment. Upon exposure to TBHP, T-AOC, CAT, GSH-Px, and T-SOD activities all significantly increased (all *p* < 0.001 vs. vector + PBS group), reflecting an adaptive response to oxidative stress. Notably, the STC-1 overexpression group showed further enhancement of T-AOC, CAT, GSH-Px, and T-SOD activities compared with the pcDNA3.1(+) + TBHP group (all *p* < 0.001).

### 3.3. STC-1 Overexpression Preserves Mitochondrial Membrane Potential (MMP)

**Figure 5:**
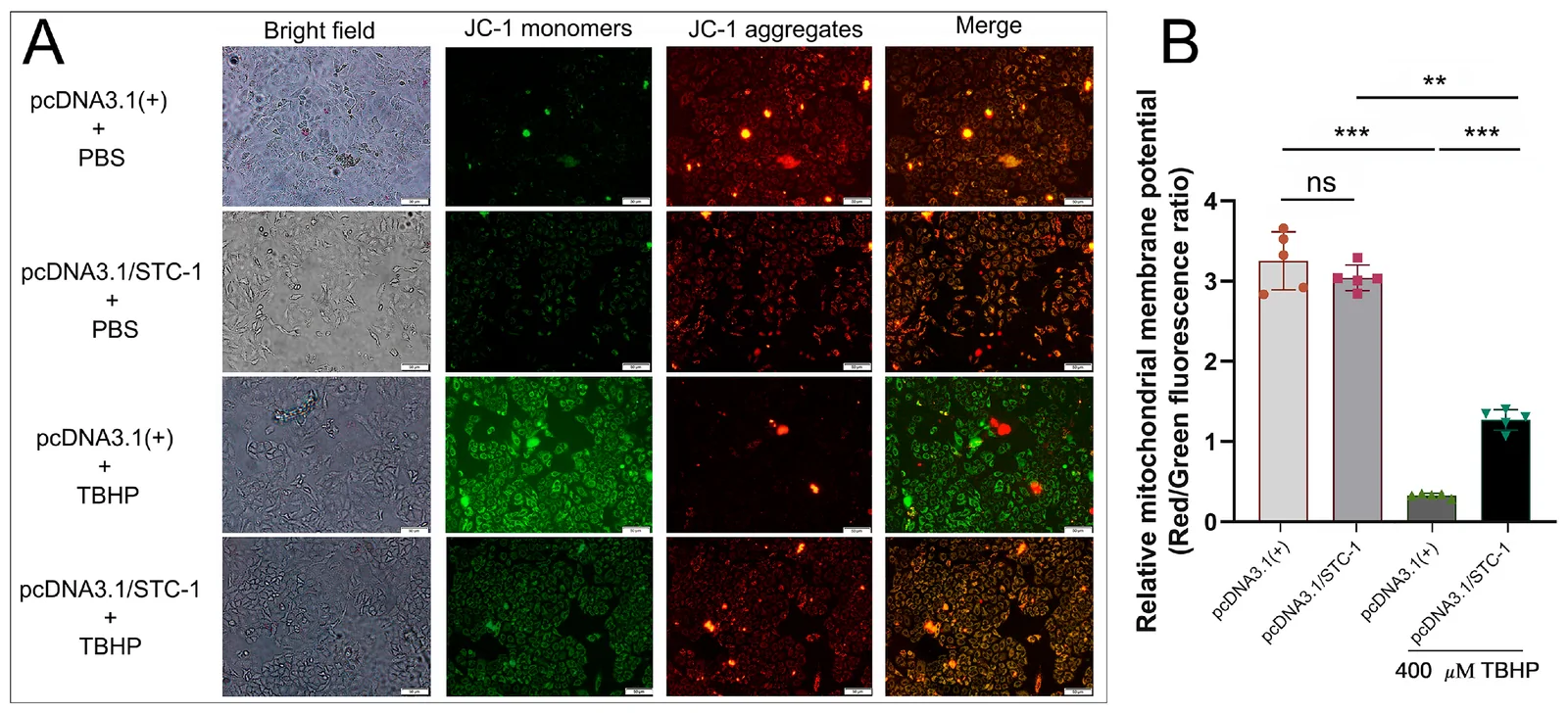
STC-1 overexpression preserves mitochondrial membrane potential (MMP) in TBHP-treated IPEC-J2 cells. Transfected cells were exposed to 200 μM TBHP for 4 h. (**A**) Representative bright-field and JC-1 fluorescence micrographs illustrating MMP changes (scale bar = 50 μm). Red fluorescence (JC-1 aggregates) indicates intact polarized mitochondria, whereas green fluorescence (JC-1 monomers) indicates depolarized mitochondria. (**B**) Quantification of the relative MMP, expressed as the ratio of red/green fluorescence intensity. Data are expressed as mean ± SD (*n* = 5). Statistical significance is indicated within the panels (** *p* < 0.01, *** *p* < 0.001; ns, *p* > 0.05).

The JC-1 staining results revealed that the transfected cells treated with PBS did not show a significant difference in MMP (Figure 5A), as evidenced the comparable red/green fluorescence ratios between the two PBS-treated groups (Figure 5B). TBHP exposure markedly decreased the red/green fluorescence ratio in pcDNA3.1(+)-transfected cells (*p* < 0.001), indicating severe mitochondrial depolarization. In contrast, STC-1 overexpression partially restored the MMP following TBHP treatment, as demonstrated the increased JC-1 aggregate (red) fluorescence, reduced JC-1 monomer (green) fluorescence, and a significantly higher red/green fluorescence ratio in the pcDNA3.1/STC-1 + TBHP group compared with the pcDNA3.1(+) + TBHP group (*p* < 0.001).

### 3.4. STC-1 Overexpression Promotes Autophagic Flux Through Activation of the Pink1/Parkin Mitophagy Pathway

**Figure 6:**
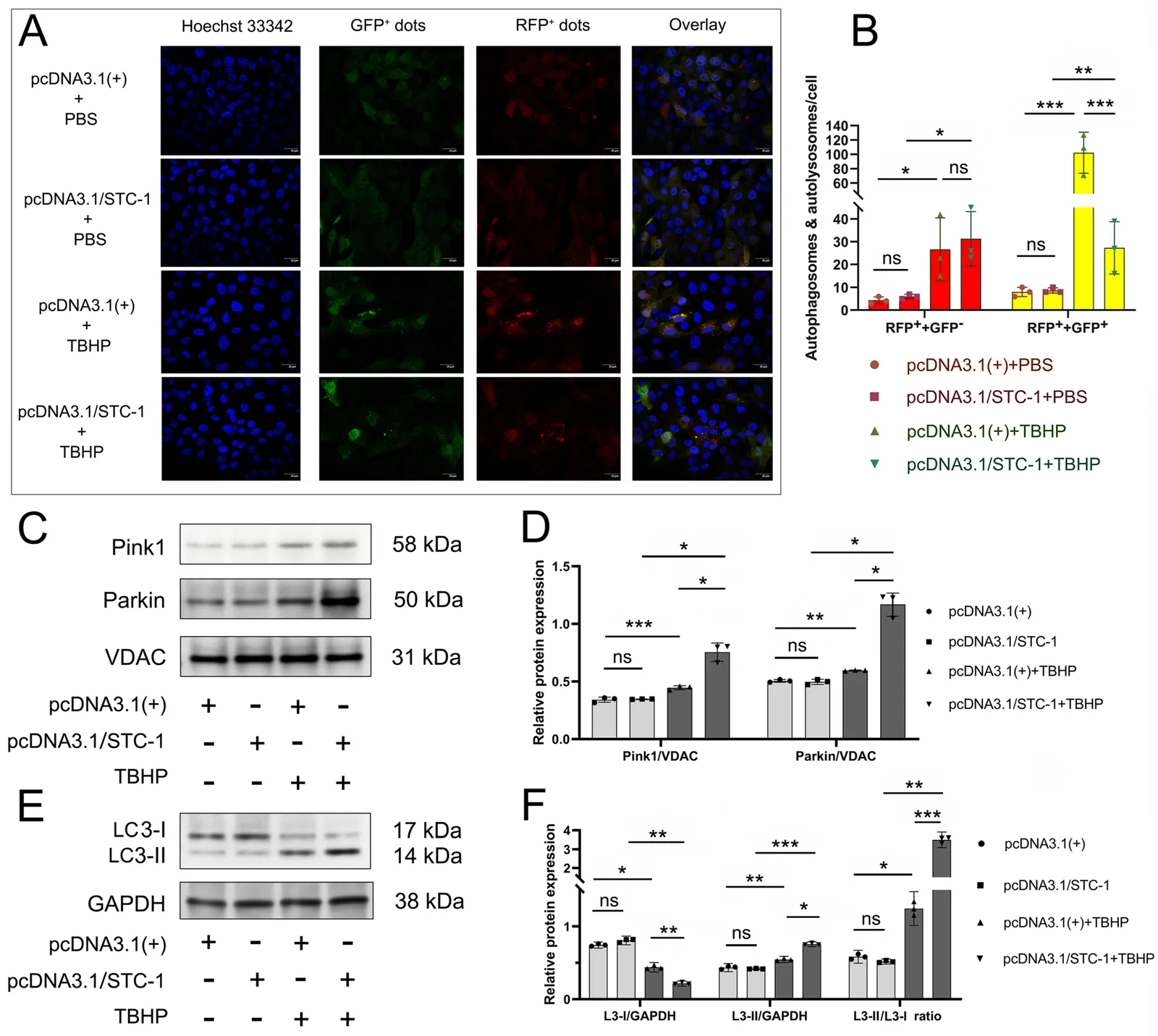
STC-1 overexpression enhances autophagic flux and activates Pink1/Parkin-mediated mitophagy in TBHP-treated IPEC-J2 cells. Cells transfected with vectors were challenged with 200 μM TBHP for 4 h. (**A**) Representative confocal fluorescence images of tandem mRFP-GFP-LC3 illustrating autophagosomes (GFP^+^/RFP^+^, yellow puncta) and autolysosomes (GFP^−^/RFP^+^, red-only puncta), with nuclei counterstained using Hoechst 33342 (scale bar = 50 μm). (**B**) Statistical quantification of autophagosomes and autolysosomes per cell. (**C**,**D**) Immunoblots and densitometric quantification of Pink1 and Parkin expression, with VDAC utilized as the mitochondrial loading control. (**E**,**F**) Immunoblots and quantification of LC3-I and LC3-II levels, including the LC3-II/LC3-I ratio, normalized to GAPDH. Data are presented as the mean ± SD (*n* = 3) of three independent experiments. * *p* < 0.05, ** *p* < 0.01, and *** *p* < 0.001; ns, *p* > 0.05 vs. the indicated group.

The cells expressing the tandem mRFP-GFP-LC3 reporter were analyzed to distinguish autophagosomes (GFP^+^/RFP^+^) from autolysosomes (GFP^−^/RFP^+^). As shown in Figure 6A,B, basal autophagic activity was comparable between the two PBS-treated groups. TBHP treatment markedly increased both autophagosomes and autolysosomes (all *p* < 0.01 vs. PBS group), indicating activation of autophagy. Compared with the pcDNA3.1(+) + TBHP group, the STC-1 overexpression group showed a significantly reduced number of autophagosomes (*p* < 0.001) while maintaining comparable numbers of autolysosomes (ns).

At the molecular level, exposure to 200 μM TBHP significantly increased Pink1 and Parkin expression (Figure 6C,D) and promoted LC3-I to LC3-II conversion (Figure 6E,F) in both transfection groups compared with the transfection + PBS groups. However, under oxidative stress conditions, the STC-1 overexpression group showed further enhancement of the expression of Pink1 and Parkin and a significantly increased LC3-II/LC3-I ratio compared with the pcDNA3.1(+) + TBHP group (all *p* < 0.01).

### 3.5. STC-1 Overexpression Is Accompanied by Upregulation of the AMPK-Nrf2/Sirt1 Signaling Axis and Promotes Expression of Mitochondrial Biogenesis-Related Proteins

**Figure 7:**
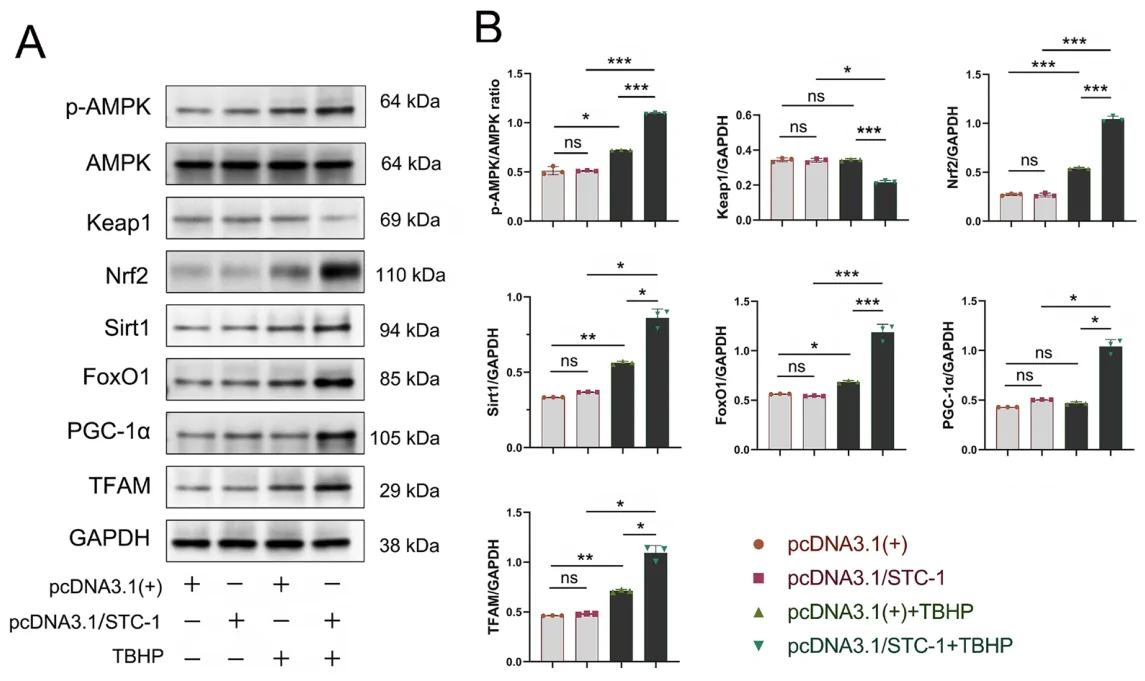
The expression levels of proteins involved in the AMPK–Nrf2/Sirt1 signaling axis and mitochondrial homeostasis were examined in TBHP-treated IPEC-J2 cells. (**A**) Representative immunoblots showing the protein expression of AMPK pathway components (p-AMPK, AMPK, Keap1, Nrf2, and Sirt1) and mitochondrial homeostasis markers (FoxO1, PGC-1α, and TFAM) in transfected IPEC-J2 cells following a 4 h challenge with 200 μM TBHP, with GAPDH as the internal loading control. (**B**) Densitometric quantification of the p-AMPK/AMPK ratio and relative protein expression levels, normalized to GAPDH. Data are presented as the mean ± SD (*n* = 3) of three independent experiments. * *p* < 0.05, ** *p* < 0.01, and *** *p* < 0.001; ns, *p* > 0.05 vs. the indicated group.

To elucidate the molecular basis of the cytoprotective effects of STC-1, the expression of key proteins involved in the AMPK-Nrf2/Sirt1 signaling pathway and mitochondrial biogenesis was analyzed via Western blotting (Figure 7A,B). TBHP treatment significantly increased the p-AMPK/AMPK ratio and the expression of Nrf2, Sirt1, FoxO1, PGC-1α, and TFAM, while decreasing Keap1 expression (all *p* < 0.05 vs. PBS treatment groups) in both transfection groups. However, compared with the pcDNA3.1(+) + TBHP group, the STC-1 overexpression group showed further enhancement of TBHP-induced activation of the AMPK/Nrf2/Sirt1 axis, which was characterized by increased AMPK phosphorylation and elevated expression of Nrf2, Sirt1, FoxO1, PGC-1α, and TFAM, together with reduced Keap1 expression (all *p* < 0.05).

## 4. Discussion

Oxidative stress is a major contributor to intestinal epithelial dysfunction and compromised gut health, particularly during the post-weaning period in piglets. Excessive accumulation of ROS disrupts epithelial barrier function, impairs cellular metabolism, and increases susceptibility to intestinal disorders [1]. Consequently, identifying mechanisms that enhance antioxidant defense and preserve mitochondrial homeostasis is important for improving intestinal stress resistance. Using a TBHP-induced oxidative stress model in IPEC-J2 cells, the present study investigated the potential protective role of STC-1 overexpression. The results suggest that STC-1 overexpression is associated with enhanced antioxidant defense, preservation of mitochondrial homeostasis, and activation of stress-responsive signaling pathways, which may collectively contribute to improved cellular adaptation to oxidative stress.

Based on our findings, the cytoprotective effects of STC-1 may be primarily associated with its ability to limit intracellular and mitochondrial ROS accumulation, thereby alleviating oxidative damage, as reflected the reduced lipid peroxidation (indicated by reduced MDA levels), nitrosative stress (indicated by decreased NO production), membrane injury (indicated by decreased LDH release), and cell death. This antioxidant effect may, at least in part, be attributed to the enhanced activities of endogenous antioxidant enzymes, including CAT, GSH-Px, and T-SOD. Consistent with this observation, STC-1 overexpression was accompanied by reduced Keap1 expression and increased Nrf2 protein levels under oxidative stress. Given the established role of the Keap1–Nrf2-ARE pathway in regulating antioxidant enzyme gene expression [24], these findings raise the possibility that activation of Nrf2 signaling contributes to the enhanced antioxidant capacity observed in STC-1-overexpressing cells. Intriguingly, STC-1 exerts these effects exclusively under oxidative challenge, leaving basal redox status unaltered, which implies that it functions as an inducible adaptive regulator.

In addition to enhancing antioxidant defenses, the reduction in ROS by STC-1 may also be related to the preservation of mitochondrial function. Mitochondria are both a major source and a primary target of oxidative stress, and mitochondrial depolarization is known to amplify ROS production and promote mitochondria-dependent apoptosis [25]. In the present study, STC-1 overexpression attenuated TBHP-induced mitochondrial membrane depolarization, reduced mitochondrial superoxide accumulation, and lowered the Bax/Bcl-2 ratio. These observations suggest that STC-1 may help preserve mitochondrial homeostasis under oxidative stress, thereby limiting the amplification of mitochondrial-derived ROS and modulating the Bax/Bcl-2 balance; these results support the possibility that maintenance of mitochondrial homeostasis is a key mechanism through which STC-1 limits oxidative stress-induced cellular damage [16,26].

Mitophagy serves as a critical mitochondrial quality control mechanism by selectively removing dysfunctional mitochondria before they become major sources of excessive ROS, NO, and pro-apoptotic signals [27]. Under oxidative stress, activation of this pathway is generally considered an adaptive response that helps maintain mitochondrial integrity and cellular survival [6]. In the present study, tandem mRFP-GFP-LC3 analysis showed that TBHP treatment increased both non-fused autophagosomes and fully matured autolysosomes, indicating activation of autophagy [8]. Notably, compared with TBHP treatment alone, STC-1 overexpression was associated with more efficient autophagic maturation rather than simple accumulation of early autophagic structures. Consistent with this observation, STC-1 overexpression was accompanied by increased Pink1 and Parkin expression as well as an elevated LC3-II/LC3-I ratio. This ensures timely engulfment of dysfunctional mitochondria by LC3-positive autophagosomes and subsequent degradation within lysosomes [9]. Although these findings do not directly demonstrate enhanced mitophagic flux, they raise the possibility that STC-1 facilitates mitochondrial quality control under oxidative stress. Such regulation may promote the clearance of damaged mitochondria, thereby limiting the accumulation of mitochondrial-derived ROS and pro-apoptotic signals. This coordinated response could contribute, at least in part, to the improved mitochondrial function and reduced cell death observed in STC-1-overexpressing cells.

Maintenance of mitochondrial homeostasis requires a balance between the removal of damaged mitochondria and the generation of new organelles. Excessive mitophagy without compensatory mitochondrial biogenesis may reduce mitochondrial content, impair energy metabolism, and compromise cell survival [28]. In the present study, STC-1 overexpression was associated with increased expression of the mitochondrial biogenesis-related proteins PGC-1α, and TFAM, which is consistent with previous reports [26,29]. PGC-1α is widely recognized as a key regulator of mitochondrial biogenesis, and TFAM is required for mitochondrial DNA replication and transcription [30,31]. Together with the observed upregulation of Pink1 and Parkin, these findings raise the possibility that STC-1 helps maintain mitochondrial homeostasis by coordinating mitochondrial turnover and renewal during oxidative stress.

The AMPK–Nrf2/Sirt1 signaling pathway may represent an important mechanism underlying these protective effects [32]. AMPK serves as a central sensor of cellular energy status and has been implicated in antioxidant responses through Nrf2 activation and regulation of Sirt1 expression [33]. As an NAD^+^-dependent deacetylase, activated Sirt1 can regulate downstream effectors, including FoxO1 and PGC-1α, which are implicated in autophagy regulation [34,35] and mitochondrial biogenesis [36] in response to oxidative stress, respectively. In the present study, STC-1 overexpression further increased the p-AMPK/AMPK ratio and the protein levels of Nrf2, Sirt1, FoxO1, PGC-1α, and TFAM under oxidative stress. Together with the enhanced mitochondrial quality control responses observed in STC-1-overexpressing cells, these findings suggest that the AMPK–Nrf2/Sirt1 axis may be involved in the STC-1-associated regulation of antioxidant defense, mitophagy, and mitochondrial biogenesis. Nevertheless, these findings do not establish a direct causal link between STC-1 and AMPK activation. STC-1 may regulate AMPK directly or indirectly through upstream metabolic or stress-responsive signals, which warrants further investigation; however, previous studies have suggested that STC-1 may directly activate the AMPK pathway in several disease models [32,37,38].

## 5. Conclusions

In conclusion, this study demonstrates that STC-1 overexpression attenuates TBHP-induced oxidative injury in porcine intestinal epithelial cells. Furthermore, the results indicate that STC-1 overexpression is associated with enhanced antioxidant defense, preserves mitochondrial function, increases the expression of proteins involved in mitophagy and mitochondrial biogenesis, and promotes the AMPK–Nrf2/Sirt1 signaling pathway, which may collectively contribute to its protective effects against oxidative stress. More importantly, this study extends the current knowledge by proposing that STC-1 may function as a regulator of mitochondrial homeostasis, integrating antioxidant defense, mitophagy, and mitochondrial biogenesis in intestinal epithelial cells. These findings provide new mechanistic insight into the role of STC-1 in oxidative stress adaptation and support its potential as a molecular target for strategies aimed at improving intestinal health and stress resilience in animal production.

## Figures and Tables

**Table 1:** Details of antibodies used in this study for Western blotting.

Antibody Name	Manufacturer	Art. No.	Dilution Ratio	Theoretical MW
STC-1 rabbit pAb ^1^	Novus Biologicals, Littleton, CO, USA	NBP1-59310	1:1000	~28 kDa
Bax rabbit pAb	Beyotime, Shanghai, China	AF0057	1: 500	~23 kDa
Bcl-2 rabbit pAb	Beyotime	AB112	1: 500	~26 kDa
Pink1 rabbit pAb	Beyotime	AF7755	1: 500	~58 kDa
Parkin rabbit pAb	Bioss, Beijing, China	bs-23687R	1: 500	~50 kDa
LC3 rabbit pAb ^2^	Sanying, Wuhan, China	14600-1-AP	1:500	~17 kDa
p-AMPKα (Thr172) rabbit mAb ^3^	Sanying	80209-6-RR	1:500	~64 kDa
AMPKα rabbit pAb	Sanying	10929-2-AP	1:1000	~64 kDa
Keap1rabbit pAb	Beyotime	AF7335	1:500	~69 kDa
Nrf2 rabbit pAb	Beyotime	AF7623	1:500	~110 kDa
Sirt1 rabbit pAb	Beyotime	AF0282	1:600	~94 kDa
FoxO1 rabbit pAb	Beyotime	AF603	1:600	~85 kDa
PGC-1α rabbit pAb	Beyotime	AF7736	1:500	~105 kDa
TFAM rabbit pAb	Beyotime	AF8127	1:500	~29 kDa
GAPDH rabbit mAb	Beyotime	AG0122	1:1000	~38 kDa
β-actin rabbit mAb	Servicebio, Wuhan, China	GB15003	1:1000	~42 kDa
VDAC rabbit mAb	Servicebio	GB151939	1:1000	~31 kDa
Goat anti-rabbit IgG-HRP	Servicebio	SAB43714	1:10,000	

^1^ Polyclonal antibody; ^2^ antibody that can cross-react with MAP1LC3A, MAP1LC3B, and MAP1LC3C; and ^3^ monoclonal antibody.

## Data Availability

The original contributions presented in this study are included in the article. Further inquiries can be directed to the corresponding author.

## Abbreviations

The following abbreviations are used in this manuscript:

STC-1	Stanniocalcin-1
TBHP	tert-butyl hydroperoxide
ROS	Reactive oxygen species
MMP	Mitochondrial membrane potential
DMSO	Dimethyl sulfoxide
CAT	Catalase
GSH-Px	Glutathione peroxidase
SOD	Superoxide dismutase
T-AOC	Total antioxidant capacity
Bax	Bcl-2-associated X protein
Bcl-2	B-cell lymphoma 2
Pink1	PTEN-induced putative kinase 1
Parkin	Parkinson disease (autosomal recessive, juvenile) 2
LC3	Microtubule-associated protein 1 light chain 3
AMPK	AMP-activated protein kinase
p-AMPK	Phospho-AMPK
Nrf2	Nuclear factor erythroid 2-related factor 2
KEAP1	Kelch-like ECH-associated protein 1
Sirt1	Sirtuin 1
FoxO1	Forkhead Box Protein O1
PGC-1α	Peroxisome proliferator-activated receptor gamma coactivator 1-alpha
TFAM	Mitochondrial transcription factor A
GAPDH	Glyceraldehyde-3-phosphate dehydrogenase
VDAC	Voltage-dependent anion channel
